# Activation of regulated cell death in the lung of piglets infected with virulent PRRSV-1 Lena strain occurs earlier and mediated by cleaved Caspase-8

**DOI:** 10.1186/s13567-020-00882-x

**Published:** 2021-01-22

**Authors:** Jose María Sánchez-Carvajal, Inés Ruedas-Torres, Librado Carrasco, Francisco José Pallarés, Enric Mateu, Irene Magdalena Rodríguez-Gómez, Jaime Gómez-Laguna

**Affiliations:** 1 Department of Anatomy and Comparative Pathology and Toxicology, Faculty of Veterinary Medicine, University of Córdoba, 14014 Córdoba, Spain; 2 Department of Animal Health and Anatomy, Faculty of Veterinary Medicine, Autonomous University of Barcelona, 08193 Bellaterra, Spain; 3 Institut de Recerca i Tecnologia Agroalimentàries - Centre de Recerca en Sanitat Animal (IRTA-CReSA), Campus de la Universitat Autònoma de Barcelona, 08193 Cerdanyola del Vallès, Spain

**Keywords:** PRRSV-1, virulent strain, regulated cell death, lung, cleaved caspase-8

## Abstract

PRRSV-1 virulent strains cause high fever, marked respiratory disease and severe lesions in lung and lymphoid organs. Regulated cell death (RCD), such as apoptosis, necroptosis and pyroptosis, is triggered by the host to interrupt viral replication eliminating infected cells, however, although it seems to play a central role in the immunopathogenesis of PRRSV, there are significant gaps regarding their sequence and activation upon PRRSV-infection. The present study evaluated RCD events by means of caspases expression in the lung of PRRSV-1-infected pigs and their impact on pulmonary macrophage subpopulations and lung lesion. Conventional piglets were intranasally inoculated with the virulent subtype 3 Lena strain or the low virulent subtype 1 3249 strain and euthanised at 1, 3, 6, 8 and 13 dpi. Lena-infected piglets showed severe and early lung damage with a high frequency of PRRSV-N-protein^+^ cells, depletion of CD163^+^ cells and high viral load in the lung. The number of TUNEL^+^ cells was significantly higher than cCasp3^+^ cells in Lena-infected piglets during the first week post-infection. cCasp8 and to a lesser extent cCasp9 were activated by both PRRSV-1 strains after one week post-infection together with a replenishment of both CD163^+^ and Arg-1^+^ pulmonary macrophages. These results highlight the induction of other forms of RCD beyond apoptosis, such as, necroptosis and pyroptosis during the first week post-infection followed by the activation of, mainly, extrinsic apoptosis during the second week post-infection. The recovery of CD163^+^ macrophages at the end of the study represents an attempt to restore pulmonary macrophage subpopulations lost during the early stages of the infection but also a macrophage polarisation into M2 macrophages.

## Introduction

During the last two decades, several outbreaks caused by virulent *Porcine reproductive and respiratory syndrome virus-1* (PRRSV-1) strains have been reported along Europe, leading to high mortality, fever, acute respiratory clinical signs as well as severe damage in both lung and primary lymphoid organs [1–6]. Lung lesion can range from the typical interstitial pneumonia observed with low virulent strains to suppurative bronchopneumonia after infection with virulent strains, usually associated with secondary bacterial infection [3, 5, 7]. In this line, although apoptosis and necrosis have been well characterised in lymphoid organs of virulent PRRSV-1-infected animals [8–11], limited information is available regarding the onset of cell death phenomena in the lung.

PRRSV encompasses two different viral species, *Betarterivirus suid 1* (formerly PRRSV-1) and *Betarterivirus suid 2* (formerly PRRSV-2) [12], and belongs to the genus *Betaarterivirus* (family *Arteriviridae*, order *Nidovirales*). PRRSV has a restricted tropism for cells of the monocyte/macrophage lineage [13], being the pulmonary alveolar macrophage (PAM) the target cell for viral replication and CD163 scavenger receptor the essential mediator for viral internalization and disassembly [14, 15]. PRRSV is also able to replicate in other pulmonary macrophage subpopulations such as interstitial and intravascular macrophages (PIMs) [13, 16, 17]. Whereas PAMs represent the first line of defence against pathogen invasion [18, 19], PIMs and interstitial macrophages play a pivotal role in the production of pro-inflammatory cytokines compromising their basic functions, including cytokine and chemokines production, polarisation, phagocytosis and antigen presentation upon PRRSV infection [20–24].

Regulated cell death (RCD) is considered a sophisticated molecular mechanism which can take place in physiological conditions, and then referred to as programmed cell death, or after extracellular or intracellular perturbations, comprising different modalities such as intrinsic and extrinsic apoptosis, necroptosis, pyroptosis and NETotic cell death, among others [25]. Thus, during the course of viral infections, RCD can be activated to interrupt viral replication and eliminate virus-infected cells [26]. Apoptosis is an evolutionary conserved mechanism of RCD that can be started by either external or internal stimuli and requires the activation of cysteine-aspartic proteases known as caspases, which results in the degradation of cellular components, DNA fragmentation and formation of apoptotic bodies, which are then mainly phagocytosed by macrophages [27–30]. In this cascade of events, caspase-8 and caspase-9 play a key role as initiator caspases, triggering off extrinsic or intrinsic apoptosis, respectively. In both cases, initiator caspases will ultimately activate the executioner caspases, such as caspase-3, for dismantling of dying cells [25, 31]. In recent years, other mechanisms of RCD, such as necroptosis or pyroptosis [25, 32], which also play a pivotal role in antiviral responses, have been well described [26, 33, 34]. Thus, when viruses try to manipulate RCD for its own advantage by inhibiting caspases-3, -8 and -9 signalling pathways, pyroptosis and necroptosis may be elicited by the host to destroy infective and non-infective cells, restricting viral replication and limiting reservoir cells for viruses [32, 33]. Thereby, there are many crosstalk between different RCD pathways and, as a result, innate immune signalling events are well activated even upon virus inhibitors [26, 34].

It is well-known that PRRSV elicits apoptosis, necrosis and necrosis like-apoptosis in lungs and lymphoid organs causing a detrimental effect of the host immune system [11, 17, 35–40]. Nevertheless, the study of RCD in the lung associated with virulent strains is scarce and just focused on detection of cell death by TUNEL [11, 41]. The objective of this study was to evaluate RCD events in the lung of pigs infected with PRRSV-1 strains of different virulence and their impact on pulmonary macrophage subpopulations as well as its contribution to lung lesion development. We hypothesised that strain dependent induction of RCD might directly impact on the impairment of the innate and adaptive immune responses at lung level, decreasing pulmonary macrophages, hence increasing the susceptibility of infected pigs to secondary infections.

## Material and methods

### Animals and experimental design

Animals and samples used in this study belong to a large project carried out in order to investigate the pathogenesis of PRRSV-1 strains of different virulence [42]. Briefly, a total of sixty-five 4-week-old piglets (Landrace x Large White) were assigned to three different experimental groups: (i) control group (n = 15), (ii) 3249-infected group (n = 25) and (iii) Lena-infected group (n = 25). All pigs included in the study were tested for relevant pathogens, such as PRRSV, porcine circovirus type 2 and *Mycoplasma hyopneumoniae* by ELISA and PCR assays (IDEXX PRRS X3 Ab Test, IDEXX Laboratories, S.L., Barcelona, Spain; in-house PCR against porcine circovirus type 2 [43] and *Mycoplasma hyopneumoniae* [44]), being negative for all of them at the beginning of the experiment. After a week of acclimation, 3249- and Lena-infected groups were intranasally inoculated with 2 ml of the low virulent 3249 strain or the virulent Lena strain (1 × 10^5^ TCID_50_) diluted in sterile RPMI 1640 medium (ThermoFisher Scientific, Barcelona Spain), respectively. The field isolate 3249 strain (subtype 1 PRRSV-1) was first isolated from the serum of a piglet with pneumonia belonging to a PRRSV-positive Spanish farm in 2005 [45]. Lena strain (subtype 3 PRRSV-1) was isolated from a PRRSV-positive herd with a high mortality rate, reproductive failure and respiratory disorders in 2007 in Belarus [3]. Piglets from control group were inoculated with porcine alveolar macrophages supernatant diluted in sterile RPMI 1640 medium (ThermoFisher Scientific) similarly to the inoculum. At 1, 3, 6, 8 and 13 days post-inoculation (dpi), 3 animals from the control group and 5 animals from both infected groups were humanely euthanised. At necropsy, gross lung lesions were recorded by the same pathologist as described elsewhere [46]. Samples from cranial, middle and caudal lobes of the right lung were collected and conserved at – 80 ºC for the analysis of PRRSV viral load. In addition, samples from the middle lobe of the right lung were fixed in 10% neutral buffered formalin for the histopathological and immunohistochemical analyses.

### PRRSV lung viral load

RNA was isolated and purified from a homogenate of lung tissue by using TRIzol™ LS Reagent and NucleoSpin® RNA virus columns kit according to manufacturer’s protocols (Macherey–Nagel, Düren, Germany). Then, viral load in the lung was quantified by RT-qPCR using VetMAX™ PRRSV EU/NA 2.0 kit (Thermo Fisher Scientific, Barcelona, Spain). RNA was amplified in the MyiQ™2 Two-Color Real-Time PCR Detection System (Bio-Rad, Hercules, CA, USA) under the following cycling conditions: 50 °C for 5 min, 95 °C for 10 min followed by 40 cycles of 95 °C for 3 s and 60 °C for 30 s. In order to not overestimate the number of PRRSV viral particles in the lung, results for viral load were expressed by changes in cycle threshold (Ct) as previously described [47, 54]. An inter-run calibrator sample with a known number of PRRSV copies was introduced in each experiment to self-control inter-run variation. The area under the curve (AUC) for viral load in the lung was calculated using the trapezoidal approach [48].

### Histopathology of lung tissue

Four-micron tissue sections were stained with haematoxylin and eosin (H&E) and blindly scored by two pathologists for histopathological evaluation. The severity of lung lesions for the interstitial pneumonia was graded according to Halbur et al. [46], as follows: no microscopic lesions, 0; mild interstitial pneumonia, 1; moderate multifocal interstitial pneumonia, 2; moderate diffuse interstitial pneumonia, 3; and severe interstitial pneumonia, 4. Moreover, a similar score was conducted as previously described by Rodríguez-Gómez et al. [42] to evaluate suppurative bronchopneumonia: no microscopic lesions, 0; mild bronchopneumonia, 1; moderate multifocal bronchopneumonia, 2; moderate diffuse bronchopneumonia, 3; and severe bronchopneumonia, 4. The sum of both interstitial pneumonia and bronchopneumonia scores were considered the final score*.*

### Immunohistochemistry assays

Terminal dUTP Nick End-Labelling (TUNEL) assay (In Situ Cell Death Detection Kit, POD, Roche, Mannheim, Germany) and cleaved Caspase-3 (cCasp3) (SignalStain Apoptosis Cleaved Caspase-3 IHC Detection Kit, Cell Signaling Technology, Inc., MA, USA) were performed by using commercial kits according to the manufacturer’s protocols. In the case of TUNEL assay, sixty-one out of sixty-five animals were selected according to lung histopathology score as detailed in Table 1.

**Table 1:** Number of pigs from each group subjected to TUNEL assay at 1, 3, 6, 8 and 13 days post-infection (dpi)

Dpi	Control	3249	Lena
1	3	4	4
3	3	5	5
6	2	5	5
8	2	5	5
13	3	5	5

3249: field strain of low virulence.

Lena: virulent PRRSV-1 strain.

The avidin–biotin-peroxidase complex technique was performed to detect PRRSV-N-protein, CD163, Arginase-1 (Arg-1), cleaved Caspase-8 (cCasp8) and cleaved Caspase-9 (cCasp9). Briefly, sections were dewaxed in xylene and rehydrated followed by endogenous peroxidase inhibition with 3% H_2_O_2_ in methanol for 30 min in the darkness. Thereafter, slides were exposed to different antigen retrieval treatments as summarised in Table 2. After PBS washes (pH 7.4) and incubation with 100 µL of 2% bovine serum albumin (BSA), monoclonal primary antibodies (mAb) against PRRSV-N-protein, CD163, Arg-1, cCasp8 and cCasp9 were incubated overnight at 4 ºC in a humid chamber. Afterwards, sections were washed with PBS and incubated with a biotinylated goat anti-mouse secondary antibody (diluted 1 in 100 in 2% BSA) (Dako, Glostrup, Denmark) for 30 min at room temperature. Then, the avidin–biotin-peroxidase complex (ABC Vector Elite, Vector Laboratories, Burlingame, CA, USA) was applied and incubated for 1 h at room temperature. Immunolabelling was visualised by application of the NovaRED™ substrate kit (Vector Laboratories, Burlingame, CA, USA). Sections were counterstained with Harris’s haematoxylin, dehydrated in graded ascending alcohols and mounted with Eukitt® (Orsatec GmbH, Bobingen, Germany). Antibody specificity was verified by substituting the primary antibody by isotype matched reagents of irrelevant specificity. For negative controls, the primary antibody was replaced by BSA blocking solution.

**Table 2:** Summary of immunohistochemical methodology

Specificity	Type of antibody	Commercial origin	Blocking solution	Dilution	Antigen retrieval
*TUNEL*	N.A	In Situ Cell Death Detection Kit, POD, Roche, Germany	N.A	N.A	Proteinase K in heat incubator
*cCasp3 (Asp175)*	mAb	SignalStain Apoptosis Cleaved Caspase-3 IHC Detection Kit, Cell Signaling, USA	5% NGS	1:500	pH 6 citrate buffer in microwave
*PRRSV (SDOW 17)*	mAb	Rural Technologies, Brookings, SD, USA	2% BSA	1:500	Protease XIV in water bath
CD163^*^ (2A10/11)	mAb	INIA, Madrid, Spain	2% BSA	Neat	pH 3.2 citrate buffer microwave
*Arginase-1*	pAb	ThermoFisher Scientific, Barcelona, Spain	2% BSA	1:100	pH 8.5 citrate buffer microwave
*cCasp8 (Asp391)*	mAb	Cell Signaling; Danvers, MA, USA	2% BSA	1:100	Proteinase K in heat incubator
*cCasp9 (Asp330)*	mAb	Cell Signaling; Danvers, MA, USA	2% BSA	1:50	Proteinase K in heat incubator

mAb: monoclonal antibody, pAb: polyclonal antibody, N.A.: not applicable, NGS: Normal goat serum, BSA: Bovine serum albumin (Sigma-Aldrich, Taufkirchen, Germany); Proteinase K (Roche, Basel, Switzerland), 15 min at 37 °C in heat incubator; Protease Type XIV (Sigma-Aldrich), 8 min at 37 °C in water bath. *CD163 mAb was kindly provided by Dr. J. Domínguez, INIA, Madrid, Spain.

The labelled cells were counted in 25 non-overlapping selected high magnification fields of 0.2 mm^2^ (Olympus BX51, Olympus Iberia SAU, L’Hospitalet de Llobregat, Barcelona, Spain). The number of positive cells per mm^2^ (cells/mm^2^) was expressed as the mean of the score for each animal within each group. Immunolabelled cells were morphologically identified as PAMs, PIMs, interstitial macrophages, lymphocytes, or neutrophils. The AUC for cCasp3, cCasp8 and cCasp9 was calculated using the trapezoidal approach [48].

### Statistical analyses

Data analyses and figures were performed by using GraphPad Prism software version 7.0 (GraphPad software, San Diego, CA, USA) and InkScape software version 0.92.3 (InkScape, Brooklyn, NY, USA). Differences between groups were evaluated for approximate normality of distribution by the D’Agostino & Pearson omnibus normality test, followed by the Mann Whitney’s U test. Correlation coefficients were assessed by the Spearman and Pearson tests and were considered relevant if r > 0.6 or r < -0.6 and *P* < 0.05. For all data, a *P* value lower than 0.05 was considered statistically significant and represented as “*”*P* ≤ 0.05, “**”*P* ≤ 0.01 and “***”*P* ≤ 0.001.

## Results

### Lena-infected pigs displayed severe lung lesions compared to 3249-infected pigs

**Figure 1:**
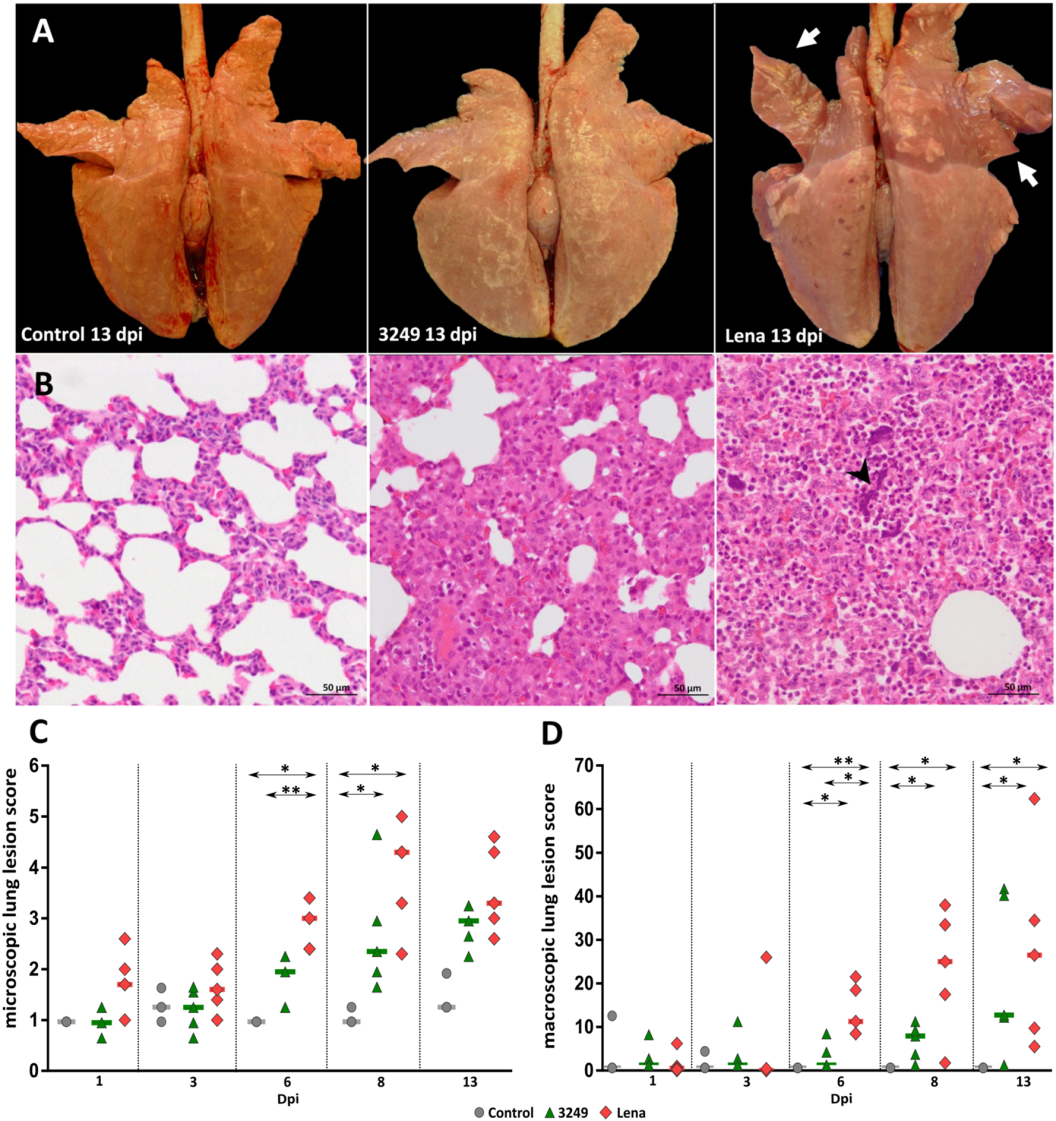
**Macroscopic and microscopic lung findings.** Pictures show the macroscopic lung appearance **A** at necropsy of representative control (left), 3249 (middle) and Lena (right) animals euthanised at 13 dpi. Lungs from 3249 (middle) and Lena (right) infected pigs shown tan-mottled areas, atelectasis, rubbery consistency, but also consolidated areas are observed in cranial apical and middle lung lobes in Lena-infected pigs (right) (white arrows). Photomicrographs of the middle lung lobe **B** from control (left), 3249 (middle) and Lena (right) pigs euthanised at 13 dpi (H&E; bars 50 μm). Lung from 3249 (middle) and Lena (right) representative infected piglets show a moderate interstitial pneumonia characterised by thickening of the alveolar septa due to infiltrating lymphocytes and macrophages. Moreover, Lena-infected lung (right) exhibits extensive foci of suppurative bronchopneumonia with neutrophils, cellular debris, and multifocal chromatin clumps (arrow-head). Graphs display macroscopic **C** and microscopic **D** lung lesion score of each animal along the study. Bars indicate median values for each group.

Gross lesions and histopathology were thoroughly described by Rodríguez-Gómez et al. [42]. At necropsy, tan-mottled areas, atelectasis, rubbery consistency, and consolidated areas were observed in the lungs from both PRRSV-1-infected groups with a marked increase from 6 dpi onwards (Figure 1A–C). From this time-point and until the end of the study, lung lesions were more intense in Lena- than in 3249-infected pigs and consisted of severe interstitial pneumonia and extensive consolidated areas in cranial and middle lung lobes (Figure 1A–C). The main histopathological lesion in the lung of both infected groups consisted of a mild to moderate interstitial pneumonia characterised by thickening of the alveolar septa due to infiltrating lymphocytes and macrophages with occasional syncytial cells (Figure 1B). This lesion had a stronger and earlier onset in pigs inoculated with virulent Lena strain (Figures 1B, C). Moreover, extensive foci of suppurative bronchopneumonia composed of neutrophils, cells debris and mucus filling the bronchial, bronchiolar, and alveolar lumen were observed from 6 dpi onwards, particularly in those animals infected with Lena strain. Multifocal clumps of purple amorphous material which were proved to be free chromatin by Feulgen technique [42], were observed in the alveoli of some of the Lena-infected pigs with higher bronchopneumonia scores.

### Lung viral load was higher in Lena-infected pigs

**Figure 2:**
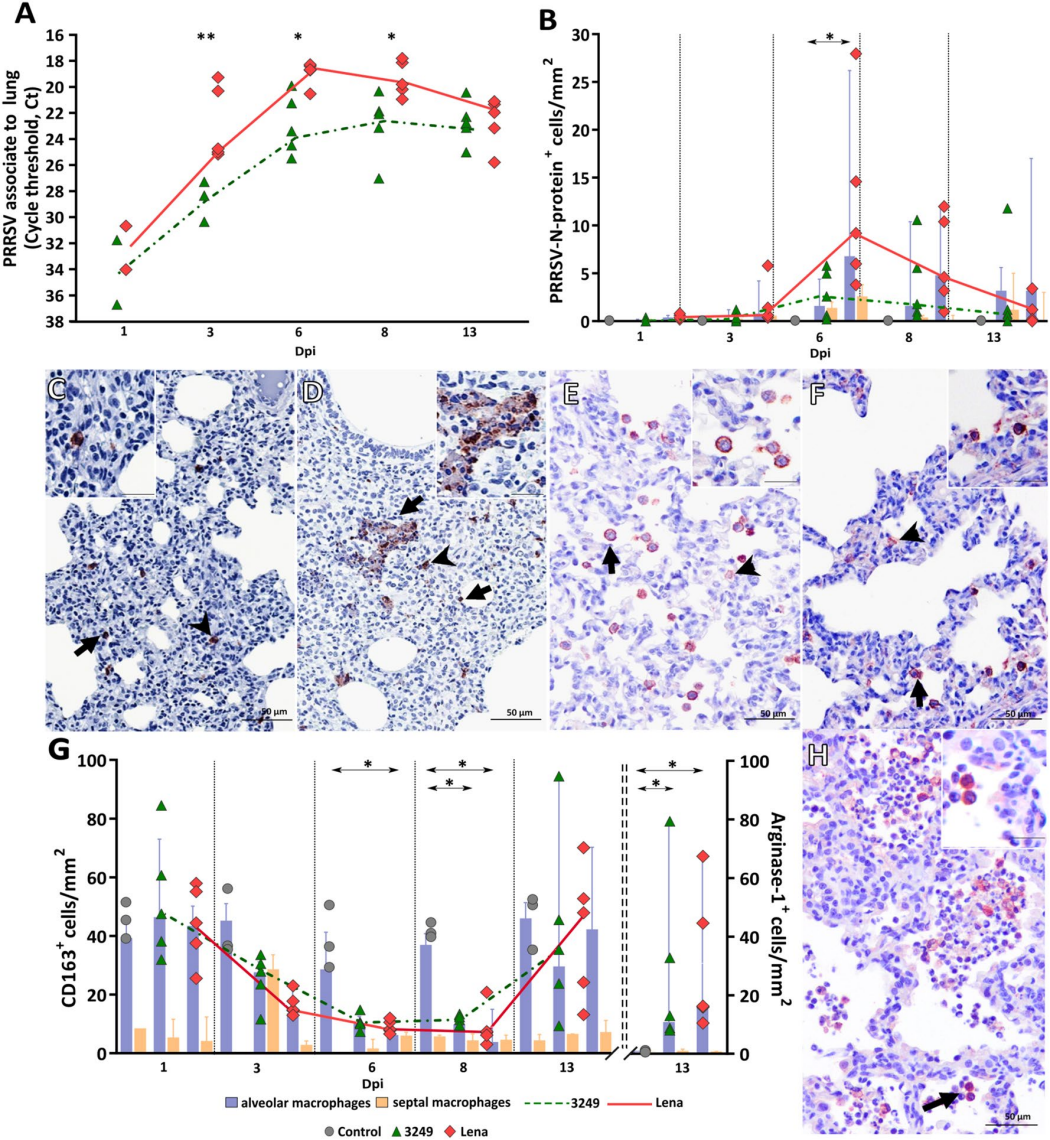
**PRRSV kinetics of viral load and immunohistochemical expression of PRRSV-N-protein, CD163 and Arg-1 in the lung.** Graphs display the kinetics of viral load in the lung for Lena and 3249 strains. Viral load is represented by changes in the cycle threshold (*C*_*t*_) **A** and the number of PRRSV-N-protein positive cells/mm^2^ **B** in the lung. Scatter dot plot shows individual values from each animal, bars and dashed lines indicate the median for each group and time points. Columns show the median with range of PAMs and septal macrophages (PIMs and interstitial macrophages). Photomicrographs of the middle lung lobe show the expression of PRRSV-N-protein in a representative 3249- **C** and Lena-infected pig **D** euthanised at 6 dpi (bar, 50 μm). Insets show in detail PRRSV-N-protein^+^ expression and viral particles (bar, 20 μm). Photomicrographs of the middle lung lobe show the expression of CD163 in a representative control **E** and Lena-infected **F** pig euthanised at 6 dpi (bar, 50 μm). Insets show in detail the immunolabelling of CD163 in the cell membrane and cytoplasm of PAMs (bar, 20 μm). Graph displays the number of CD163^+^ (primary axis) and Arg-1^+^ (secondary axis) cells/mm^2^ in the lung for each group and time point (**G**). Scatter dot plots show individual values from each animal, bars and dashed lines indicate the median for each group and time point. Columns represent the median with range of PAMs and septal macrophages. Photomicrograph of the middle lung lobe show the expression of Arg-1^+^ PAMs in a representative Lena-infected pig at 13 dpi **H** (bar, 50 μm). Inset shows in detail Arg-1 expression in PAMs (bar, 20 μm). Black arrows represent PAMs, and black arrow-heads interstitial macrophages.

At day 0, all animals were negative by RT-qPCR, remaining pigs from control group negative throughout the study. As early as 1 dpi, two out of five piglets in both infected groups were PRRSV-1 positive; from this time-point onwards, viral copies were detected in all infected piglets. Viral load was higher in Lena- compared to 3249-infected pigs (*P* < 0.01 at 3 dpi; *P* < 0.05 at 6 and 8 dpi), reaching the peak at 6 dpi in Lena (*C*_*t*_ 18.9 ± 0.9), whereas 3249 group peaked at 8 dpi (*C*_*t*_ 22.9 ± 2.5). At the end of the study, viral load in the lung was comparable in both infected groups, Lena and 3249 (Figure 2A). The AUC for viral load in lung (median) was 300.0 and 261.2 for Lena and 3249 groups, respectively. In both infected groups, statistical analysis revealed a positive significant correlation between lung viral load and the microscopic score (r = 0.7 and *P* < 0.001, for 3249 group; r = 0.6 and *P* < 0.01; for Lena group).

### *Lena-infected pigs presented the highest number of PRRSV-N-protein*^+^*cells and the lowest number of CD163*^+^*cells*

Figure 2 shows the expression of PRRSV-N-protein in the lung of each experimental group along the infection. The immunolabelling of PRRSV-N-protein was mainly observed in PAMs and to a lesser extent in PIMs and interstitial macrophages (Figures 2C, D). Multifocal clusters of PRRSV-N-protein^+^ macrophages surrounded by apoptotic bodies were observed within areas of bronchopneumonia in Lena-infected piglets (Figure 2D). Both Lena- and 3249-infected groups followed a similar kinetics for PRRSV-N-protein^+^ cells, reaching a peak at 6 dpi. At 13 dpi, the frequency of PRRSV-N-protein^+^ cells markedly dropped in both infected groups. The highest number of PRRSV-N-protein^+^ cells was observed in lung tissue from Lena-infected piglets at 6 dpi, showing a significant increase when compared to 3249-infected pigs (*P* < 0.05) (Figure 2B). No positive cells were detected in control pigs throughout the experiment (Figure 2B). A significant statistical correlation between PRRSV-N-protein expression and lung viral load was found in both infected groups (r = 0.6 and *P* < 0.01, for 3249 group; and r = 0.8 and *P* < 0.001, for Lena group).

The expression of the scavenger receptor CD163 was detected in the cell membrane and cytoplasm of PAMs, interstitial macrophages and, occasionally, PIMs (Figure 2E, F). Control animals exhibited a steady frequency of CD163^+^ cells during the whole study, whereas there was a continuous drop in the number of CD163^+^ cells from the beginning until 8 dpi, followed by a sharp rise in the frequency of CD163^+^ cells at 13 dpi in both PRRSV-1-infected groups (Figure 2G). The decrease was more intense in Lena-infected animals when compared to 3249 (Figure 2G). Of note, PAMs were the subpopulation of pulmonary macrophages which underwent the greatest reduction in the number of CD163^+^ cells. Moreover, there was a significant negative correlation between PRRSV-N-protein^+^ and CD163^+^ cells (r = -0.6 and *P* < 0.0005) in Lena-infected piglets.

To confirm macrophage polarisation into M2-alternative activated macrophages at 13 dpi, Arg-1, a M2 macrophage marker, was performed. Arg-1 immunolabelling was detected mainly in the cytoplasm of PAMs placed inside or surrounding bronchopneumonia foci, but also in PIMs and interstitial macrophages in a lesser extent (Figure 2H). There was a significantly higher frequency of Arg-1^+^ macrophages in both Lena and 3249-infected groups when compared with control group at 13 dpi (Figure 2G).

### TUNEL labelling was highly expressed in the lung of Lena-infected animals

**Figure 3:**
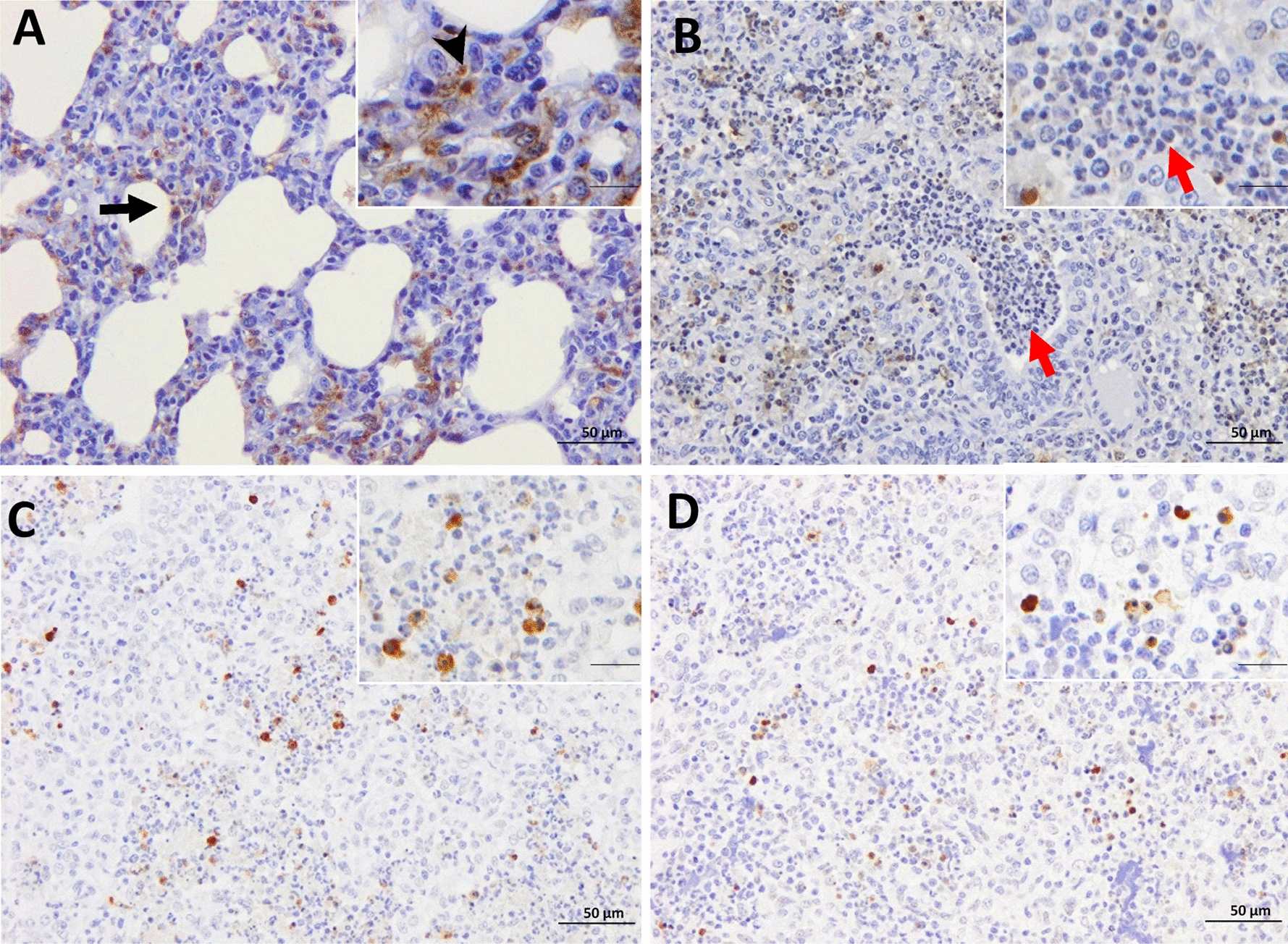
**Immunohistochemistry for TUNEL and cCasp3 in the lung.** TUNEL expression in a representative 3249- **A** and Lena-infected pig **B** euthanised at 8 dpi (bar, 50 μm). Insets show in detail the immunolabelling of TUNEL in the nuclei and cytoplasm (bar, 20 μm). PAMs (black arrow), interstitial macrophages (black arrow-head) as well as TUNEL^−^ neutrophils (red arrows). cCasp3 expression in a representative 3249- **C** and Lena-infected pig **D** euthanised at 13 dpi (bar, 50 μm). Insets show in detail the expression of cCasp3 in the nuclei and cytoplasm of PAMs (bar, 20 μm).

**Figure 4:**
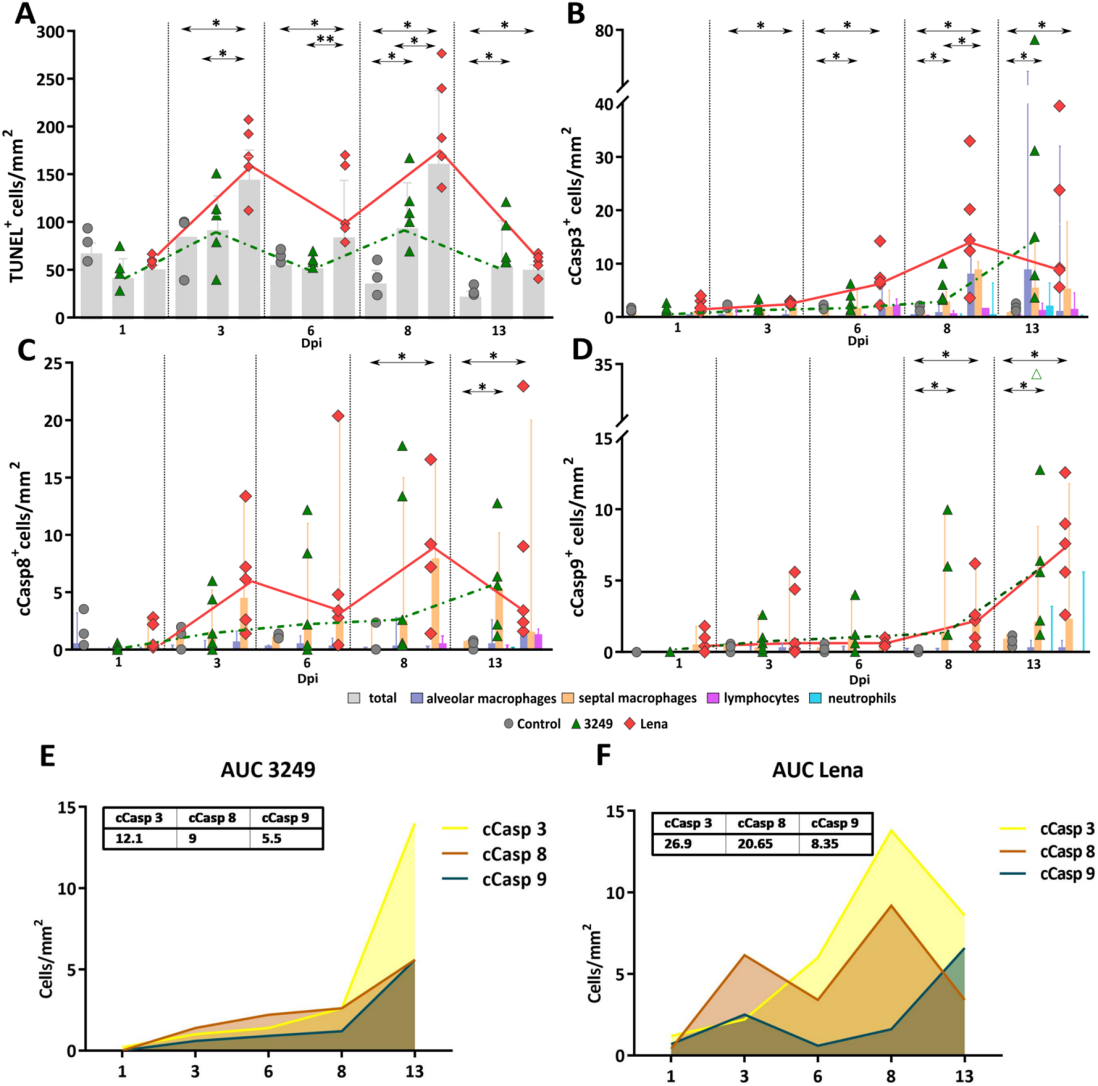
**Immunohistochemical expression of TUNEL, cCasp3, cCasp8 and cCasp9 in the lung along the experiment.** Graph displays the number of TUNEL^+^ in the lung (cells/mm^2^) (**A**). Scatter dot plots show individual values from each animal and dashed lines indicate the median for each group and time points. Columns represent the median with range of total positive cells. Graph represents the number of cCasp3^+^ (**B**), cCasp8^+^ (**C**) cCasp9^+^ (**D**) in the lung (cells/mm^2^). Scatter dot plots show individual values from each animal and dashed lines indicate the median for each group and time point. Columns represent the median with range of PAMs, septal macrophages lymphocytes and neutrophils. One pig infected with 3249 strain at 6 dpi was considered an outlier (empty green triangle). Graph displays the AUC (median) for the frequency of cCasp3, cCasp8 and cCasp9 positive cells in 3249 **E** and Lena-infected **F** group.

TUNEL assay was conducted to detect in situ localization of DNA fragmentation. TUNEL labelling was detected in the nuclei and cytoplasm of PAMs, PIMs, interstitial macrophages, free apoptotic bodies and, occasionally, neutrophils, in both control and infected groups (Figures 3A, B). TUNEL^+^ interstitial macrophages and PIMs were mainly observed in areas of interstitial pneumonia (Figure 3A) whereas TUNEL^+^ PAMs were identified in foci of suppurative bronchopneumonia with cell debris and mucus filling the lumen of bronchioles and alveoli (Figure 3B). However, due to the aggregation of TUNEL^+^ cells, mostly in areas of bronchopneumonia, it was not always possible to differentiate subsets of labelled cells, thus, in this case the results are expressed as the total number of TUNEL^+^ cells. The frequency of TUNEL^+^ cells was higher in Lena-infected pigs than in control animals from 3 dpi onwards (*P* < 0.05), as well as compared with 3249-infected pigs at 3 (*P* < 0.05), 6 (*P* < 0.01), and 8 dpi (*P* < 0.05) (Figure 4A). For 3249-infected pigs, the rise in TUNEL^+^ cells took place later at 8 and 13 dpi, as compared to control pigs (*P* < 0.05) (Figure 4A). The highest expression was detected at 8 dpi in both PRRSV-1 infected groups. At 13 dpi a marked dropped in the total number of TUNEL^+^ cells, but still higher than control animals, was observed in both infected groups. A negative correlation was revealed among TUNEL^+^ cells and CD163^+^ cells (r = -0.6 and *P* < 0.001) in Lena group.

### Casp3 followed a similar kinetics in Lena- and 3249-infected groups, being more intense for Lena group

cCasp3 was detected in the nuclei and cytoplasm of macrophages, lymphocytes, neutrophils and apoptotic bodies of both control and infected piglets (Figs. 3C-3D). cCasp3^+^ interstitial macrophages, PIMs and in lesser extent lymphocytes were mainly located in areas of interstitial pneumonia, whereas cCasp3^+^ PAMs and neutrophils were commonly observed infiltrating foci of bronchopneumonia (Figure 3D). The kinetics of cCasp3^+^ expression was similar in both PRRSV-1-infected groups, rising gradually from 3 dpi (Lena) or 6 dpi (3249) until the end of the study and showing a significant increase when compared to control group (*P* < 0.05 at 3, 6, 8 and 13 dpi) (Figure 4B). This increase in cCasp3 cells was earlier, as above mentioned, and more intense in Lena-infected pigs showing a peak at 8 dpi, whereas the maximum expression in 3249-infected pigs was observed at 13 dpi (Figure 4B). By contrast, the number of cCasp3^+^ cells remained at low levels in the control group along the study. In 3249-infected group, a positive correlation was found between cCasp3 expression and viral load in the lung (r = 0.6 and *P* < 0.01) as well as, between cCasp3^+^ cells and PRRSV-N-protein^+^ cells (r = 0.7 and *P* < 0.001). The AUC for cCasp3 (median) in 3249 and Lena groups was 12.1 and 26.9, respectively (Figure 4E, F).

### *A positive correlation between the frequency of cCasp8*^+^*cells and cCasp9*^+^*cells was observed in both PRRSV-1 infected groups*

**Figure 5:**
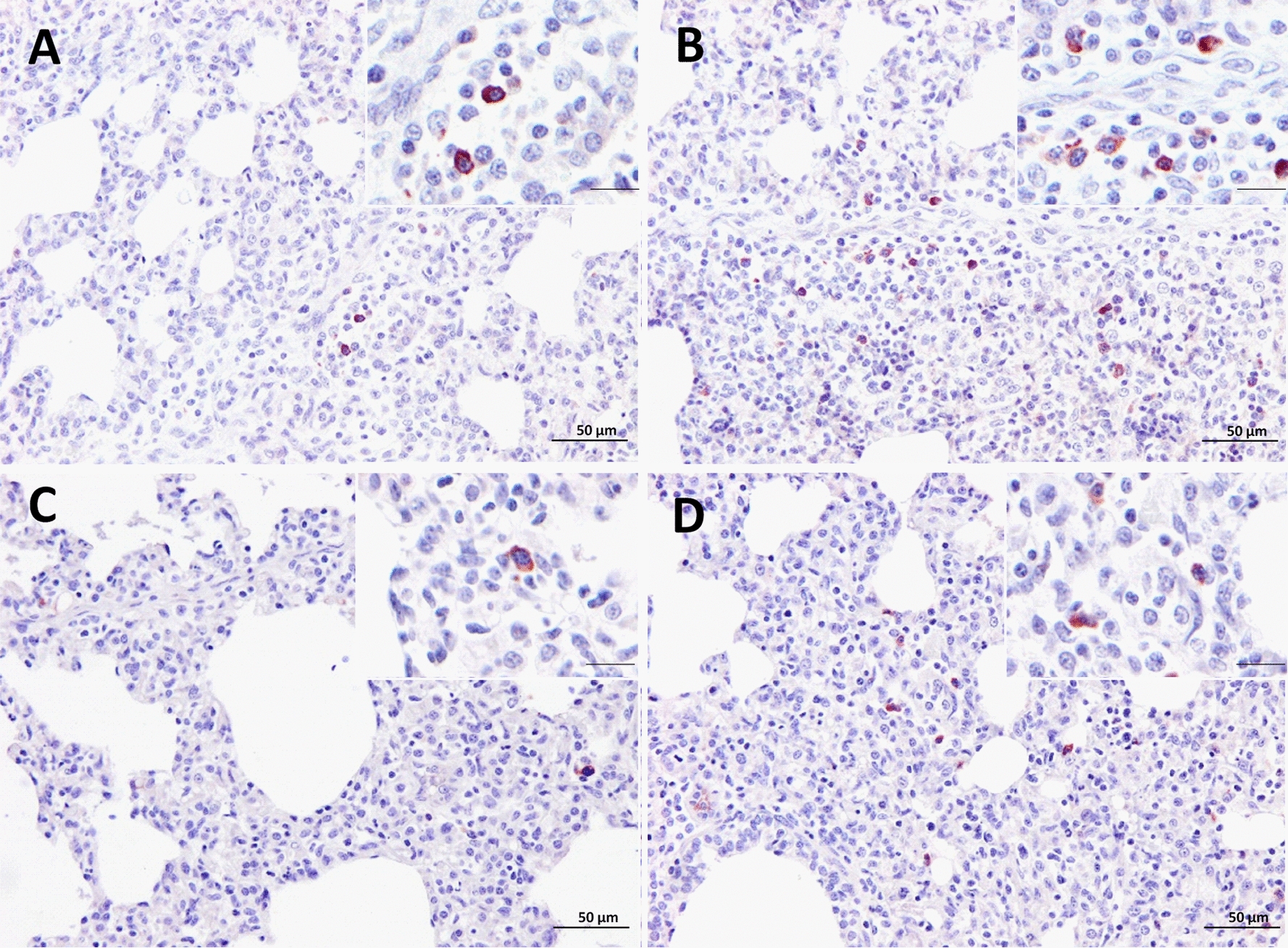
**Immunohistochemistry for cCasp8 and cCasp9 in lung tissue.** cCasp8 expression in a representative 3249- **A** and Lena-infected pig **B** euthanised at 8 dpi (bar, 50 μm). Inset shows in detail the cCasp8 immunolabelling in the cytoplasm of interstitial macrophages (bar, 20 μm)**.** cCasp9 expression in a representative 3249- **C** and Lena-infected pig **D** euthanised at 13 dpi (bar, 50 μm). Inset shows in detail the expression of cCasp9 in the cytoplasm of interstitial macrophages (bar, 20 μm).

The immunolabelling of cCasp8 was primarily detected in the cytoplasm of interstitial macrophages as well as PIMs, and in a lesser extent in PAMs and lymphocytes (Figures 5A, B). The frequency of cCasp8^+^ cells in Lena-infected pigs, showed a curve with a progressive increase, reaching a peak at 8 dpi and decreasing onwards (Figure 4C). This rise was significantly higher when compared with the control group at 8 and 13 dpi (*P* < 0.05). By contrast, the number of cCasp8^+^ cells in 3249 group remained at baseline or below control group with a significant ascent at 13 dpi (*P* < 0.05) (Figure 4C). A wide individual variability was observed in both, Lena- and 3249-infected pigs.

Regarding cCasp9, its expression was mainly observed in the cytoplasm of interstitial macrophages and, occasionally, PIMs and PAMs (Figures 5C, D). Both, PRRSV-1-infected groups displayed a similar kinetics for cCasp9^+^ cells. Thus, there was a mild gradual rise from 8 dpi until the end of the study, reaching maximum frequencies at 13 dpi in both infected groups (*P* < 0.05) (Figure 4D). Lena virulent strain induced a higher number of cCasp9^+^ cells compared to 3249 strain at 8 and 13 dpi. One pig infected with 3249 strain at 6 dpi was considered an outlier due to an extreme number of cCasp9^+^ cells (Figure 4D). The frequencies of cCasp8^+^ and cCasp9^+^ cells remained low and constant in control pigs during the whole study. In both PRRSV-1 infected groups, a positive correlation was found between the number of cCasp8^+^ cells and the number of cCasp9^+^ cells (r = 0.86 and *P* < 0.001, for 3249 group; and, r = 0.6 and *P* < 0.01, for Lena group). In addition, a positive correlation was observed in 3249-infected animals between the frequency of cCasp9^+^ cells and lung viral load (r = 0.6 and *P* < 0.01), PRRSV-N-protein^+^ cells (r = 0.6 and *P* < 0.001) and cCasp3^+^ cells (r = 0.7 and *P* < 0.001). The AUC for cCasp8 (median) in 3249 and Lena groups was 9 and 20.6, respectively, whereas for cCasp9 (median) was 5.5 in 3249 and 8.3 in Lena groups.

## Discussion

RCD is a key player in the innate response to combat viral infection, disrupting replication and destroying infected cells from the host [26]. Therefore, PRRSV, as well as many other viruses, have developed strategies to obstruct or delay apoptosis in order to obtain a window of time in which viral replication, self-assembly and release can take place [26, 34, 35, 49]. It has been showed that PRRSV-induced apoptosis is reliant on virulence-strain since PRRSV-1 virulent strains cause apoptosis and necrosis with a higher severity in lymphoid organs than low virulent strains [9–11]. However, the mechanisms involved in RCD phenomena used by virulent strains in the lung of PRRSV-1 infected pigs have not been elucidated yet.

In the present study, a marked depletion in the number of CD163^+^ PAMs in the lung of infected pigs was observed, which may lead to a failure in the phagocytosis by these cells [7] and hence, affect the clearance of apoptotic bodies. Whereas in a previous study we were able to describe the replenishment of CD163^+^ cells in the lung of SU1-bel infected pigs about one month post infection [50], this phenomenon was already observed at two weeks post-infection in the present study. This recovery of CD163^+^ PAMs, together with an increase of Arg-1^+^ macrophages, coincided with a decrease in the number of PRRSV-N-protein^+^ cells and viral load in the lung, which may depict an attempt to remove apoptotic cells and cellular debris, restoring tissue damage. Besides Arg-1 upregulation in M2-alternative activated macrophages [51, 52], the induction of CD163 is, by far, a feature of this subset, which could be playing a relevant role in tissue wound repair, accelerating the resolution of inflammation [15, 53, 54].

Subsequently, TUNEL assay was carried out to identify in situ DNA fragmentation, a feature of both apoptotic as well as necrotic cells [55]. Besides, a cCasp3 immunolabelling method was used to demonstrate the activation of the caspase-dependent apoptotic pathway. In our study, the number of TUNEL^+^ cells was significantly higher than the number of cCasp3^+^ cells. This fact was observed not only in PRRSV-1-infected but also in non-infected piglets throughout the study. Thus, this result suggests that an independent cCasp3 pathway of RCD could be involved in both pulmonary cell homeostasis of pigs and clearance of virus-infected cells, since infected pigs exhibited higher frequencies of TUNEL^+^ cells than control animals. Apoptosis, programmed necrosis (necroptosis) and pyroptosis are considered as distinct approaches of RCD, depending on the signalling pathway engaged. Apoptosis is a non-lytic and immunologically silent manner of cell death; by contrast, programmed necrosis and pyroptosis are lytic and highly inflammatory processes [25, 56]. In vitro studies have evidenced the activation of anti-apoptotic pathways in PAMs and susceptible cell lines during the early infection with PRRSV, followed by the stimulation of apoptosis in late infection [35, 49, 57]. Accordingly, our results highlight a delayed activation of apoptosis up to one week post-infection as a mechanism by which PRRSV could evade the local host immune response, but also suggest that other forms of RCD, such as necroptosis and pyroptosis, could be playing a role during the first week post-infection.

Pyroptosis may be initiated by inflammasomes, which in addition trigger the secretion of proinflammatory cytokines [58]. PRRSV is able to activate NLRP3 inflammasome in PAMs, resulting in the secretion of IL-1β and a robust inflammatory response [59–61], which are associated with a severe pathologic lung damage in piglets infected with PRRSV-1 virulent strains, such as Lena and SU1-bel strains [6, 7, 62]. In the present study, a significant rise in TUNEL^+^ cells was observed in both PRRSV-1-infected pigs, although more pronounced in Lena-infected pigs, from the beginning of the study (3 dpi). This fact may be probably associated with the cytopathic effect of PRRSV, but also with the strong inflammatory response induced in the lung, especially when the extent of lung injury was greater (8 dpi). Previously, in a parallel study, we demonstrated that Lena-infected animals developed a strong inflammatory response associated with a high serum level of IL-6 and IFN-γ, contributing to fever and more severe clinical signs [63]. In this sense, further studies will help to elucidate the link between the inflammatory response and RCD phenomena in the lung along PRRSV infection. Consequently, the activation of RCD upon virulent PRRSV-1 infection might play a double-edged sword, in one way, trying to kill faster and earlier infected cells but on the other way also causing severe lung injury.

Curiously, nets of free chromatin were observed in the alveoli of some Lena-infected pigs, accompanied by a moderate neutrophil infiltrate in areas of suppurative bronchopneumonia. Many viruses, bacteria and fungi can stimulate the formation of neutrophils extracellular traps (NETs) in a process called NETosis, another type of RCD [25, 64–69]. These net-like structures are composed of chromatin and equipped with granule protein trapping and/or killing extracellular and intracellular microorganisms to prevent their local spreading [67]; however, bacteria can also take advantage of NETs as growth source [64, 66]. Since NETs and NET precursor neutrophils are described to be TUNEL^−^ [70], and most neutrophils as well as the clumps of free chromatin observed in our study were TUNEL^−^ and cCasp3^−^, we hypothesised that these clumps could be associated with NETs triggered by neutrophils in foci of suppurative bronchopneumonia. NETs formation during infection with virulent PRRSV strains might be linked with the higher extent of the lung damage as well as with the onset of secondary bacterial infections and the porcine respiratory disease complex.

There is mounting evidence of viruses activating extrinsic and/or intrinsic apoptotic pathways [26]. In the present study, cCasp8 and cCasp9 were activated by Lena and 3249 strains after one week post-infection showing a similar kinetics and a correlation among both caspases, which is consistent with the crosstalk between extrinsic and intrinsic apoptosis pathways already described in PRRSV infection [49, 71]. Whereas Lena virulent strain induced a higher number of cCasp8^+^ cells than cCasp9^+^ cells, for 3249 strain, the expression of both caspases was quite similar. These results suggest that Lena virulent strain is able to induce a higher activation of cCasp8^+^ in the lung, which is in agreement with a recent publication of our group which describes an unidirectional activation of apoptosis in the thymus of PRRSV-1 infected pigs through the activation of the extrinsic pathway induced by Fas/cCasp8 [9]. Interestingly, different proinflammatory cytokines may play a significant role not only in the inflammatory response but also modulating apoptosis, underlining the role of TNF-α as a mediator of the extrinsic pathway of apoptosis [27, 28, 30]. In this context, our findings highlight the interest of examining different target organs and biomarkers to better understand the modulation of the host immune response.

The immunolabelling of PRRSV-N-protein was observed in PAMs and to a lesser extent in interstitial macrophages and PIMs. By contrast, apoptotic markers such as cCasp3, cCasp8 and cCasp9, were mainly observed in interstitial macrophages, PIMs, or lymphocytes located in lung interstitium and secondly in PAMs. Thus, whereas apoptosis was mainly activated in interstitial macrophages and PIMs, other mechanisms of RCD besides apoptosis, such as necroptosis and/or pyroptosis, might be involved in the cell death of PAMs. The enhancement of apoptosis phenomena in non-infected cells from the lung could be addressed by two different scenarios: (i) during PRRSV infection there is a strong influx of interstitial macrophages and monocytes mediated by cytokines and chemotactic factors in an attempt to replace the lost cell population [21, 72, 73], whose overwhelming production may lead to an excessive infiltration of cells and hence apoptosis may be triggered as homeostatic mechanism to control cell population; (ii) apoptosis of uninfected bystander cells is a key element of PRRSV infection to destroy host immune cells [36, 41, 60], which have been associated with the release of pro-inflammatory cytokines, such as TNF-α, IL-1α, IL-1β or IL-6, in the lung of pigs infected with both low virulent and virulent PRRSV-1 strains [6, 21, 36, 62] as well as other porcine viruses [74].

Taken together, the present study evaluated RCD in the lung upon infection with two PRRSV-1 strains of different virulence. Our results suggest the activation of non-apoptotic RCD during the first week post-infection followed by the activation of mainly extrinsic apoptosis, and in a lesser extent intrinsic apoptosis, during the second week post-infection. These phenomena were markedly elicited by Lena strain accompanied by a marked inflammatory response, high viral load, and severe depletion of CD163^+^ PAMs, causing severe and early lung damage. Recovery of CD163^+^ PAMs, together with an increase of Arg-1^+^ PAMs, was observed at two weeks post-infection which point out that pulmonary macrophages were polarised to M2, but also, an attempt to reconstitute the pulmonary macrophages subpopulations lost during the early stages of the infection. Further studies should be performed to decipher the role of other pathways and mediators of RCD during PRRSV infection.

## Data Availability

The datasets analysed during the current study, supporting their conclusions, are included within the article.
